# Impact of varying degree of donor-recipient weight mismatch on survival outcomes in pediatric heart transplantation

**DOI:** 10.1007/s12055-026-02195-8

**Published:** 2026-02-28

**Authors:** Rohit Ganduboina, John Karamichalis, Xander Jacquemyn, Michel Pompeu Sá, Leonardo Mulinari, Sandeep Sainathan

**Affiliations:** 1 Division of Cardiology, Cedar-Sinai Medical Center, Los Angeles, CA USA; 2 Department of Surgery, NRI Institute of Medical Sciences, Visakhapatnam, India; 3 Section of Pediatric and Congenital Cardiac Surgery, Columbia University Medical Center, New York, NY USA; 4 Department of Cardiothoracic Surgery, University of Pittsburgh, Pittsburgh, PA USA; 5 Division of Cardiothoracic Surgery, Massachusetts General Hospital, Boston, USA; 6 Division of Cardiothoracic Surgery, University of Miami, Miami, USA; 7 Department of Congenital Cardiac Surgery, Corewell Health, Helen Devos Children’s Hospital, Michigan State University, Michigan, USA

**Keywords:** Donor-recipient weight mismatch, Pediatric heart transplantation, Survival outcomes

## Abstract

**Objectives:**

Body weight is still the most common metric used for donor-recipient matching in Pediatric Heart Transplantation (PHTX) and its impact on long-term outcomes remains unclear from variable definition and conflicting evidence. In this study, the outcomes of varying weight mismatch on PHTX was analysed.

**Methods:**

The United Network for Organ Sharing (UNOS) database (1984–2025) was retrospectively analysed. Size mismatch as a percentage donor-recipient weight difference was categorized as Mild (≤ 20%), Moderate (20–30%), Extreme (> 30%), and stratified as undersizing or oversizing. Demographics, clinical characteristics, and post-transplant outcomes were compared. One-year and 15-year mortality were analysed using multivariable logistic and Cox regression, respectively.

**Results:**

Oversizing (80%, 9,175/11,583) was more common than undersizing (20%, 2,408/11,583). With increasing oversizing, the recipients were younger, had congenital heart disease (CHD) diagnosis, and elevated pulmonary vascular resistance but with shorter wait-list times. Oversizing had no impact on 30-day, 1-year, or 15-years survival. With increasing undersizing, recipient age was similar, had less restrictive cardiomyopathy diagnosis, and had female donor but with shorter wait-list times. Undersizing had inferior survival till 5-years but not at 15-years. However, on multivariable analysis, undersizing was not predictive of inferior survival anytime as was congenital heart disease diagnosis, Extracorporeal Membrane Oxygenation (ECMO) bridge, elevated pulmonary-vascular resistance, transplantation era, and post-transplant rejection, stroke, and dialysis use.

**Conclusions:**

Oversizing is more likely than undersizing in PHTX. While oversizing had no overall survival impact, undersizing had negative mid-term but not long-term survival impact which disappeared when adjusted for underlying cardiac diagnosis, elevated pulmonary vascular resistance, transplantation era and occurrence of post-transplant complications.

**Supplementary Information:**

The online version contains supplementary material available at https://doi.org/10.1007/s12055-026-02195-8.

## Introduction

Pediatric heart transplantation (PHTX) remains the definitive therapy for children with end-stage heart failure, predominantly resulting from congenital heart disease or cardiomyopathy [1]. The lack of suitable donor organs remains a major obstacle, frequently resulting in longer waitlist times and higher waitlist mortality rates [2, 3].

Traditionally, the primary metric of size compatibility in pediatric heart transplantation has been donor-recipient weight ratio (DRWR) [4, 5]. While a number of alternative indices based on anthropometrics, and imaging data have been described to better match donor to recipients, weight based matching still remains the most common method due to familiarity, unavailability of certain donor data to calculate the aforementioned indices [6, 7].

Donor-recipient size mismatch can influence outcomes beyond the immediate postoperative period. While modest oversizing, especially in infant recipients appears to be well tolerated or even helpful, undersized grafts have been linked with increased risks of graft failure, early rejection, and mortality [8]. Though DRWR is still the clinical norm, rigorous adherence to limited thresholds may unnecessarily restrict donor use, particularly for high acuity candidates on the wait-list. The evidence already in publication is still conflicting: Mismatch has been shown to have negative effects in certain subgroups (such as patients with mechanical circulatory support or elevated pulmonary vascular resistance), but other studies find no discernible long-term effects on survival [8, 9]. Small sample sizes, inconsistent mismatch definitions, and inadequate risk adjustment usually attribute for these discrepancies.

In this study, we examine the relationship between donor-recipient size mismatch and post–pediatric heart transplant outcomes, aiming to clarify the clinical significance of size disparity and determine whether observed outcomes are independently attributable to mismatch or confounded by recipient characteristics. Although alternative measures such estimated total cardiac volume have shown better physiological relevance and predictive value in some populations, as previously alluded, their use is limited in large retrospective datasets due to the lack of uniformly available imaging and anatomical data. In contrast, DRWR is universally recorded, clinically familiar, and readily applicable for large-scale risk stratification. By categorizing the severity of weight mismatch, we seek to assess its independent prognostic impact and contribute to a more evidence-based understanding of acceptable donor-recipient size thresholds in pediatric transplantation.

## Materials and methods

### Data source and study population

We conducted a retrospective cohort study using data from the Organ Procurement and Transplantation Network (OPTN), United Network for Organ Sharing (UNOS) database, including all pediatric primary heart transplants performed in the United States between 1984 and 2025. Pediatric recipients were defined as individuals under 18 years of age undergoing primary, isolated heart transplantation. Patients were excluded if key data were missing or implausible, including donor or recipient weight and recipient age. After applying these criteria, the final study population included 11,583 pediatric heart transplant recipients.

### Mismatch definition and derived metrics

Donor-recipient size compatibility was quantified using the relative weight difference, calculated as the absolute value of the difference between recipient and donor weight divided by the recipient’s weight and expressed as a percentage. This value reflects the extent of undersizing or oversizing, irrespective of direction. Recipients were classified into three categories based on this metric: Mild Mismatch (≤ 20% difference), Moderate Mismatch (20–30%), and Extreme Mismatch (> 30%).

**Fig. 1:**
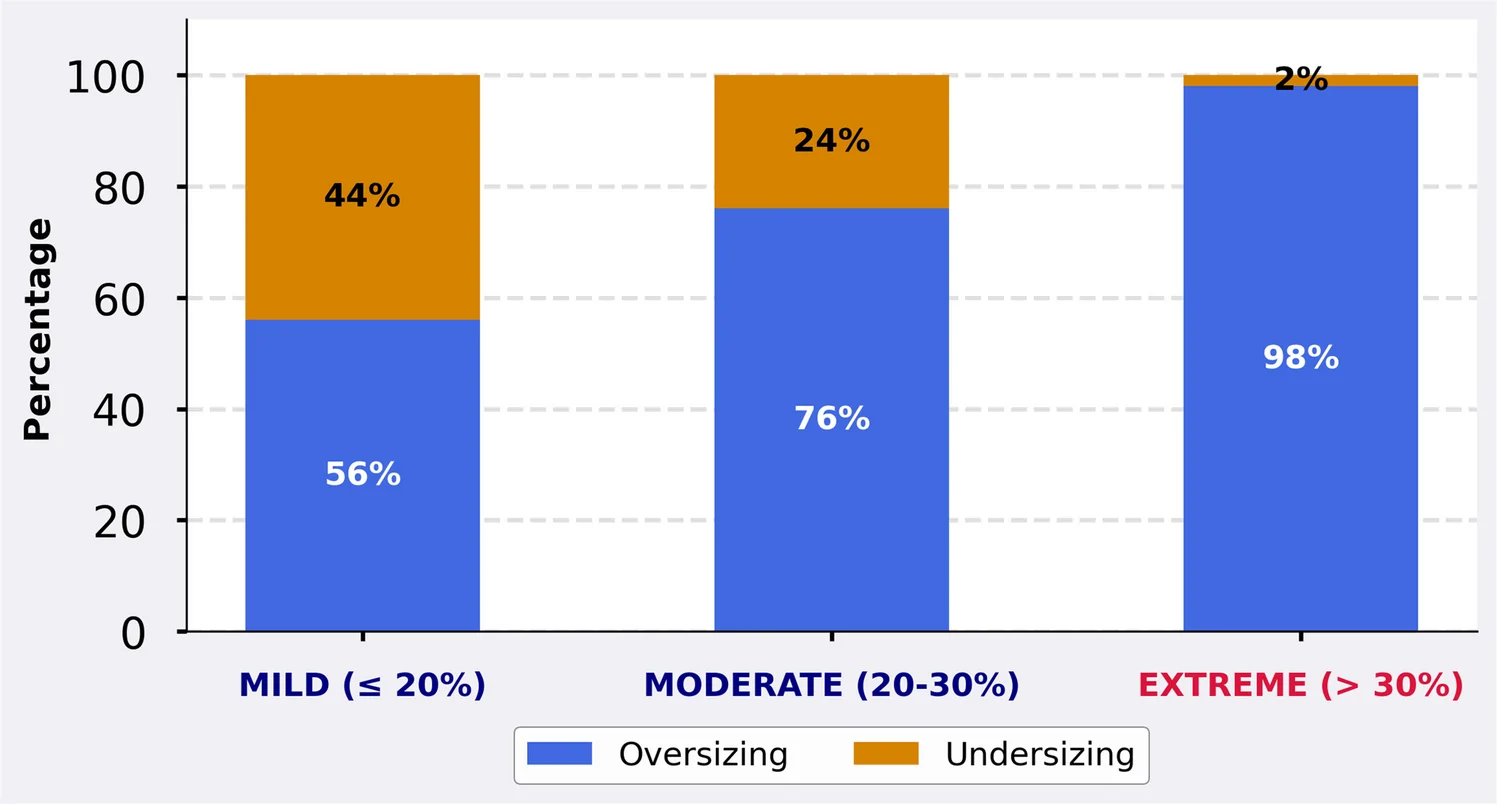
Distribution of weight disparity among pediatric heart TX

To distinguish the direction of mismatch, recipients were further divided into two groups: undersizing (donor weight less than recipient weight) and oversizing (donor weight greater than recipient weight). Within the oversizing group, 2,415 recipients had a Mild mismatch, 1,118 had a Moderate mismatch, and 5,642 had an Extreme mismatch. For the undersizing group, 1,914 recipients had a Mild mismatch, 352 had a Moderate mismatch, and 142 had an Extreme mismatch (Fig. 1). This approach allowed for the assessment of mismatch magnitude while accounting for directional effects, enabling separate analyses of undersized and oversized transplants across these three strata.

### Variables and outcomes

Extracted variables included demographic factors (recipient age, sex, race, ethnicity, blood type, and transplant era), clinical status at listing, renal and hepatic function, ischemic time, and waitlist status. Post-transplant complications such as dialysis, stroke, pacemaker placement, and late graft rejection were also recorded. Survival status was determined at multiple time points, including 30 days, 1 year, 5 years, 10 years, and 15 years post-transplant.

The primary endpoints were early mortality (1-year post-transplant mortality) and late mortality (15 years post-transplant mortality). Early mortality was analyzed using multivariable logistic regression, while late mortality was assessed using Cox proportional hazards regression.

### Statistical analysis

Descriptive statistics were computed using Python (version 3.11) with the pandas, NumPy, and SciPy libraries. Analyses were performed using complete-case data; observations with missing values in any of the variables under consideration were excluded. No imputation methods were applied. Continuous variables were summarized using means with standard deviations or medians with interquartile ranges, as appropriate. Hemodynamic measures, including Pulmonary Vascular Resistance (PVR) and Transpulmonary Gradient (TPG), were reported as medians with minimum and maximum values. Categorical variables were reported as frequencies and percentages. Comparisons across mismatch groups were made using the Kruskal–Wallis tests for continuous variables and chi-squared or Fisher exact tests for categorical variables, depending on distribution and group size.

Multivariable regression analyses were performed in R (version 4.4.0). Variable selection for multivariable modeling was based on univariate statistical testing and clinical relevance. Candidate covariates were assessed using appropriate tests based on data type and distribution. Variables with a univariate p-value less than 0.05 were selected for inclusion in the multivariable logistic and Cox proportional hazards model. Mismatch was modeled both categorically and continuously using ordinal scores (1 for Mild, 2 for Moderate, 3 for Extreme) to assess linear and quadratic trends. Inclusion of both trend terms allowed for evaluation of non-linear associations, enabling detection of potential U-shaped or J-shaped risk patterns across the Mismatch spectrum. Survival differences were visualized using Kaplan–Meier curves and compared with the log-rank test. Hazard ratios (HR) and odds ratios (OR) were reported with corresponding 95% confidence intervals (CI). Long-term survival was assessed using Cox proportional hazards regression. A two-tailed *P* value < 0.05 was considered statistically significant for all analyses.

## Results

### Baseline characteristics

#### Oversizing

Baseline characteristics differed significantly across oversizing severity groups (Table 1). Recipients in the extreme oversizing group (donor > 30% heavier; n = 5,642) were younger (median age 2.0 years [IQR 0.0–10.0]) compared to moderate (7.0 [1.0–14.0]) and mild (8.0 [1.0–14.0]) mismatch groups (*p* < 0.001). They also had lower body weight and BMI at transplant (*p* < 0.001). The median weight disparity across oversizing categories was 10.22% in the mild group, 25.00% in the moderate group, and 66.15% in the extreme group, with a maximum mismatch of 480%. Congenital heart disease (CHD) was more common in the extreme group (53.3%), contributing to significant differences in heart failure etiology across groups (*p* < 0.001). Donors in this group were younger (median age 5.0 years [1.0–14.0]; *p* < 0.001), and rates of height mismatch (> ± 5%) and ABO incompatibility were significantly higher (81.7% and 6.2%, respectively; *p* < 0.001).

**Table 1:** Transplant characteristics and demographics

Variable	Oversizing (0.01%—480.46%)				Undersizing (0%—79.84%)			
	Mild (≤ 20%) *n* = 2415, 26.3%	Moderate (20–30%) *n* = 1118, 12.2%	Extreme (> 30%) *n* = 5642, 61.5%	*P*-value	Mild (≤ 20%) *n* = 1914, 79.5%	Moderate (20–30%) *n* = 352, 14.6%	Extreme (> 30%) *n* = 142, 5.9%	*P*-value
Weight Disparity (%) [Median (Range)]	10.22 (0.01–20.00)	25.00 (20.00–30.00)	66.15 (30.00–480.46)	< 0.001	7.89 (0.0–20.00)	23.84 (20.10–30.00)	36.55(30.13–79.84)	< 0.001
Recipient Characteristics								
Age (years)	8.00 [1.00–14.00]	7.00 [1.00–14.00]	2.00 [0.00–10.00]	< 0.001	8.00 [1.00–14.00]	7.00 [0.00–15.00]	8.50 [0.00–14.75]	0.417
Female	1048 (43.4%)	481 (43.0%)	2514 (44.6%)	0.475	808 (42.2%)	148 (42.0%)	57 (40.1%)	0.890
Race				0.003				0.536
Caucasian	1367 (56.6%)	612 (54.7%)	3237 (57.4%)		1062 (55.5%)	196 (55.7%)	71 (50.0%)	
African American	487 (20.2%)	240 (21.5%)	985 (17.5%)		401 (21.0%)	82 (23.3%)	40 (28.2%)	
Hispanic	426 (17.6%)	196 (17.5%)	1046 (18.5%)		329 (17.2%)	59 (16.8%)	22 (15.5%)	
Asian	72 (3.0%)	31 (2.8%)	229 (4.1%)		69 (3.6%)	11 (3.1%)	4 (2.8%)	
Other	60 (2.5%)	35 (3.1%)	133 (2.4%)		51 (2.7%)	4 (1.1%)	5 (3.5%)	
Body Mass Index (kg/m2)	17.53 [15.19–21.21]	17.24 [15.12–20.00]	15.80 [14.14–17.78]	< 0.001	17.98 [15.38–23.91]	18.81 [16.05–28.10]	21.15 [16.69–31.74]	< 0.001
Blood Type				0.664				0.774
O	1060 (43.9%)	512 (45.8%)	2547 (45.1%)		887 (46.3%)	167 (47.4%)	68 (47.9%)	
B	333 (13.8%)	141 (12.6%)	718 (12.7%)		242 (12.6%)	45 (12.8%)	23 (16.2%)	
A	915 (37.9%)	426 (38.1%)	2140 (37.9%)		705 (36.8%)	122 (34.7%)	45 (31.7%)	
AB	107 (4.4%)	39 (3.5%)	237 (4.2%)		80 (4.2%)	18 (5.1%)	6 (4.2%)	
Heart Failure Etiology				< 0.001				0.029
Congenital Heart Disease	1094 (45.3%)	523 (46.8%)	3009 (53.3%)		813 (42.5%)	145 (41.2%)	58 (40.8%)	
Restrictive Cardiomyopathy	241 (10.0%)	105 (9.4%)	421 (7.5%)		167 (8.7%)	38 (10.8%)	6 (4.2%)	
Dilated Cardiomyopathy	981 (40.6%)	445 (39.8%)	2031 (36.0%)		867 (45.3%)	161 (45.7%)	66 (46.5%)	
Valvular	10 (0.4%)	3 (0.3%)	18 (0.3%)		6 (0.3%)	2 (0.6%)	1 (0.7%)	
Ischemic	4 (0.2%)	2 (0.2%)	15 (0.3%)		5 (0.3%)	0 (0.0%)	0 (0.0%)	
Other	85 (3.5%)	40 (3.6%)	148 (2.6%)		56 (2.9%)	6 (1.7%)	11 (7.7%)	
Cerebrovascular accident prior transplant	73 (3.0%)	29 (2.6%)	138 (2.4%)	0.434	52 (2.7%)	9 (2.6%)	3 (2.1%)	0.977
Prior cardiac surgery	119 (4.9%)	47 (4.2%)	278 (4.9%)	0.790	69 (3.6%)	11 (3.1%)	7 (4.9%)	0.430
ECMO Bridge	97 (4.0%)	39 (3.5%)	279 (4.9%)	0.038	82 (4.3%)	21 (6.0%)	6 (4.2%)	0.372
Recipient on VAD	288 (11.9%)	151 (13.5%)	569 (10.1%)	< 0.001	216 (11.3%)	27 (7.7%)	12 (8.5%)	0.089
Prior Dialysis History	52 (2.2%)	31 (2.8%)	129 (2.3%)	0.523	44 (2.3%)	5 (1.4%)	4 (2.8%)	0.474
Total bilirubin (mg/dL)	0.60 [0.40–1.10]	0.70 [0.40–1.20]	0.60 [0.40–1.20]	0.298	0.60 [0.40–1.10]	0.60 [0.30–1.20]	0.60 [0.40–0.90]	0.546
Serum creatinine (mg/dL)	0.50 [0.32–0.70]	0.50 [0.30–0.70]	0.40 [0.30–0.60]	< 0.001	0.50 [0.30–0.71]	0.60 [0.34–0.80]	0.60 [0.40–0.90]	0.005
Positive CMV serology	748 (31.0%)	355 (31.8%)	1570 (27.8%)	0.291	578 (30.2%)	99 (28.1%)	33 (23.2%)	0.546
Cardiac index (L/min/m2) [mean ± SD]	3.12 ± 1.93	3.25 ± 2.13	3.62 ± 2.79	< 0.001	3.09 ± 2.29	2.73 ± 1.88	2.49 ± 1.53	0.003
Mean pulmonary artery pressure (mm Hg) [mean ± SD]	24.86 ± 11.23	24.63 ± 11.00	25.04 ± 11.71	0.959	25.27 ± 11.19	25.95 ± 11.17	27.73 ± 12.16	0.171
Mean pulmonary capillary wedge pressure (mm Hg) [mean ± SD]	16.60 ± 8.14	16.28 ± 7.36	15.76 ± 7.46	0.014	16.95 ± 8.38	17.28 ± 8.05	17.65 ± 8.73	0.688
Pulmonary vascular resistance (Woods units) [Median (min – max)]	2.40 (0.13–98.00)	2.31 (0.18–62.22)	3.04 (0.10–115.00)	< 0.001	2.31 (0.10–32.14)	2.19 (0.30–48.65)	2.17 (0.23–27.66)	0.946
Trans-pulmonary gradient (mm Hg) [Median (min – max)]	7.00 (1.00–73.00)	7.00 (1.00–57.00)	8.00 (1.00–59.00)	0.001	7.00 (1.00–58.00)	7.00 (1.00–68.70)	8.00 (1.00–40.00)	0.292
Donor Characteristics								
Age (years)	7.00 [1.00–16.00]	7.00 [1.00–17.00]	5.00 [1.00–14.00]	< 0.001	6.00 [0.00–16.00]	5.00 [0.00–16.00]	5.50 [0.00–15.00]	0.051
Female	969 (40.1%)	448 (40.1%)	2315 (41.0%)	0.681	760 (39.7%)	166 (47.2%)	65 (45.8%)	0.017
Race				0.036				0.312
Caucasian	1379 (57.1%)	650 (58.1%)	3141 (55.7%)		1089 (56.9%)	220 (62.5%)	90 (63.4%)	
African American	521 (21.6%)	234 (20.9%)	1190 (21.1%)		397 (20.7%)	65 (18.5%)	33 (23.2%)	
Hispanic	419 (17.3%)	199 (17.8%)	1153 (20.4%)		367 (19.2%)	60 (17.0%)	17 (12.0%)	
Asian	49 (2.0%)	19 (1.7%)	87 (1.5%)		31 (1.6%)	4 (1.1%)	0 (0.0%)	
Other	43 (1.8%)	15 (1.3%)	65 (1.2%)		29 (1.5%)	3 (0.9%)	2 (1.4%)	
Body mass index (kg/m2)	18.55 [15.87–22.23]	18.94 [16.13–22.37]	18.56 [16.12–21.97]	0.253	18.19 [15.55–22.03]	17.26 [14.54–20.94]	16.29 [13.44–20.70]	< 0.001
Blood type				0.210				0.642
O	1426 (59.0%)	671 (60.0%)	3260 (57.8%)		1151 (60.1%)	217 (61.6%)	92 (64.8%)	
B	226 (9.4%)	99 (8.9%)	502 (8.9%)		163 (8.5%)	29 (8.2%)	14 (9.9%)	
A	729 (30.2%)	336 (30.1%)	1770 (31.4%)		579 (30.3%)	104 (29.5%)	36 (25.4%)	
AB	34 (1.4%)	12 (1.1%)	110 (1.9%)		21 (1.1%)	2 (0.6%)	0 (0.0%)	
Mechanism of death				0.224				0.352
Head trauma	1193 (49.4%)	575 (51.4%)	2805 (49.7%)		991 (51.8%)	167 (47.4%)	70 (49.3%)	
Anoxia	886 (36.7%)	387 (34.6%)	2033 (36.0%)		624 (32.6%)	129 (36.6%)	41 (28.9%)	
CVA	197 (8.2%)	68 (6.1%)	418 (7.4%)		170 (8.9%)	27 (7.7%)	17 (12.0%)	
CNS tumor	16 (0.7%)	8 (0.7%)	35 (0.6%)		15 (0.8%)	2 (0.6%)	1 (0.7%)	
Other	120 (5.0%)	79 (7.1%)	346 (6.1%)		114 (6.0%)	27 (7.7%)	13 (9.2%)	
Mismatch and Transplant Characteristics								
Gender Mismatch	1125 (46.6%)	533 (47.7%)	2731 (48.4%)	0.323	844 (44.1%)	150 (42.6%)	70 (49.3%)	0.394
Height Mismatch (> ± 5%)	1333 (55.2%)	697 (62.3%)	4611 (81.7%)	< 0.001	1041 (54.4%)	202 (57.4%)	82 (57.7%)	0.465
Donor Weight (kg)	26.90 [10.50–59.00]	26.90 [10.43–60.30]	20.00 [11.00–52.70]	< 0.001	22.00 [8.17–59.25]	18.00 [5.27–55.08]	20.20 [4.00–54.00]	< 0.001
Recipient Weight (kg)	24.00 [9.70–53.90]	21.20 [8.43–48.97]	12.00 [6.10–30.40]	< 0.001	23.50 [9.08–63.58]	23.85 [7.14–74.03]	35.25 [6.98–85.70]	0.501
ABO incompatibility				< 0.001				0.317
Compatible	2328 (96.4%)	1076 (96.2%)	5290 (93.8%)		1839 (96.1%)	344 (97.7%)	137 (96.5%)	
Incompatible	87 (3.6%)	42 (3.8%)	352 (6.2%)		75 (3.9%)	8 (2.3%)	5 (3.5%)	
HLA mismatch (> 3 loci mismatch)	1796 (74.4%)	806 (72.1%)	4022 (71.3%)	0.018	1372 (71.7%)	237 (67.3%)	106 (74.6%)	0.164
Donor distance to transplanting center (nautical miles)	292.00 [119.75–444.00]	299.00 [120.00–460.50]	305.00 [130.00–473.00]	0.008	281.00 [102.00–450.00]	290.00 [93.00–474.00]	317.00 [125.00–496.00]	0.123
Ischemic Time (hours)	3.58 [2.93–4.20]	3.57 [2.92–4.20]	3.62 [2.97–4.28]	0.026	3.53 [2.88–4.18]	3.56 [2.92–4.20]	3.58 [2.98–4.35]	0.637
Heart Listing Status at the time of transplant								
Before Allocation Change (n)	527	188	1245	< 0.001	449	110	69	< 0.001
Old Status 1	243 (46%)	99 (53%)	807 (65%)		230 (51%)	71 (64%)	50 (72%)	
Status 2	284 (54%)	89 (47%)	438 (35%)		219 (49%)	39 (36%)	19 (28%)	
After Allocation Change (n)	863	445	2026	0.001	613	79	28	< 0.001
Status 1A	676 (78%)	367 (82%)	1701 (84%)		499 (81%)	63 (80%)	26 (92.9%)	
Status 1B	157 (18%)	71 (16%)	277 (14%)		96 (16%)	13 (16%)	1 (3.6%)	
Status 2	30 (4%)	7 (2%)	48 (2%)		18 (3%)	3 (4%)	1 (3.6%)	
Transplantation year	2012.00 [2003.00–2018.00]	2013.00 [2004.00–2019.00]	2011.00 [2001.00–2019.00]	< 0.001	2011.00 [2001.00–2018.00]	2007.00 [1998.00–2015.00]	2003.00 [1995.00–2013.00]	< 0.001
Transplantation Era				< 0.001				< 0.001
1987–1997	343 (14.2%)	127 (11.4%)	989 (17.5%)		305 (15.9%)	77 (21.9%)	57 (40.1%)	
1998–2007	529 (21.9%)	234 (20.9%)	1292 (22.9%)		442 (23.1%)	101 (28.7%)	32 (22.5%)	
2008–2017	837 (34.7%)	399 (35.7%)	1678 (29.7%)		677 (35.4%)	109 (31.0%)	33 (23.2%)	
2018–2025	706 (29.2%)	358 (32.0%)	1683 (29.8%)		490 (25.6%)	65 (18.5%)	20 (14.1%)	
Days on Waitlist (median [IQR])	55.00 [17.00–139.00]	50.00 [18.00–123.00]	46.00 [17.00–112.00]	< 0.001	53.00 [19.00–127.00]	46.00 [15.00–104.00]	39.50 [16.25–81.50]	0.028
Post-transplant outcomes								
30 Day Survival	2301 (95.3%)	1070 (95.7%)	5377 (95.3%)	0.829	1832 (95.7%)	330 (93.8%)	124 (87.3%)	< 0.001
90 Day Survival	2253 (93.3%)	1053 (94.2%)	5223 (92.6%)	0.119	1783 (93.2%)	324 (92.0%)	121 (85.2%)	0.002
1 Year Survival	2175 (90.1%)	989 (88.5%)	4999 (88.6%)	0.135	1722 (90.0%)	314 (89.2%)	113 (79.6%)	< 0.001
5 Year Survival	1983 (82.1%)	898 (80.3%)	4530 (80.3%)	0.151	1550 (81.0%)	281 (79.8%)	98 (69.0%)	0.003
Treated Acute Rejection- 1 year	437 (18.1%)	200 (17.9%)	848 (15.0%)	0.042	375 (19.6%)	72 (20.5%)	31 (21.8%)	0.020
Dialysis	147 (6.1%)	70 (6.3%)	307 (5.4%)	0.543	118 (6.2%)	13 (3.7%)	11 (7.7%)	0.093
Stroke	66 (2.7%)	27 (2.4%)	195 (3.5%)	0.043	53 (2.8%)	3 (0.9%)	4 (2.8%)	0.105
Permanent Pacemaker	15 (0.6%)	13 (1.2%)	46 (0.8%)	0.248	16 (0.8%)	2 (0.6%)	1 (0.7%)	0.888
Hospital Length of Stay (days)	18.00 [12.00–33.00]	19.00 [13.00–33.00]	20.00 [13.00–35.00]	0.007	19.00 [12.00–35.00]	18.00 [12.00–33.00]	22.00 [14.00–33.00]	0.392

Continuous variables were compared using the Kruskal–Wallis test; categorical variables were compared using Chi-square or Fisher’s exact tests, as appropriate

Pre-transplant hemodynamic parameters also varied by oversizing severity (Table 1). Cardiac index increased with mismatch severity (extreme: 3.62 ± 2.79 vs. mild: 3.12 ± 1.93 L/min/m^2^; *p* < 0.001). The extreme group had lower pulmonary capillary wedge pressures (p = 0.014) and higher pulmonary vascular resistance (Median 3.04, Woods units; *p* < 0.001) and transpulmonary gradient (Median 8 mmHg, p = 0.001), indicating elevated pulmonary arterial pressure independent of the higher cardiac output. The Extracorporeal Membrane Oxygenation (ECMO) bridge was not different between the groups but Ventricular Assist Device (VAD) utilization as a bridge to transplant was lower in the extreme group. The donor hospital to transplant hospital distance was higher in the extreme oversize group as were the ischemic times (p < 0.05). The wait-list times were times were lower in the extreme mismatch group. While the extreme group enjoyed higher listing status with older listing status, this was not the case with the newer listing status (Table 1).

Survival outcomes at 30 days, 90 days, 1 year, and 5 years were comparable across oversizing groups (*p* > 0.05; Table 1). However, the extreme group had lower rates of treated acute rejection at 1 year (15.0% vs. 17.9–18.1%; *p* = 0.042), but higher stroke incidence (3.5% vs. 2.4–2.7%; *p* = 0.043). Median hospital length of stay increased with mismatch severity (*p* = 0.007).

#### Undersizing

In the undersizing cohort, recipient age did not differ significantly across groups (*p* = 0.417), though BMI was highest in the extreme group (Table 1). Conversely, the BMI was the lowest in the donors in the extreme mismatch group. Median weight disparity in the undersizing groups was 7.89% in the mild category, 23.84% in the moderate category, and 36.55% in the extreme category, with a maximum mismatch of 79.84%. Heart failure etiology varied by mismatch severity (*p* = 0.029), with restrictive cardiomyopathy being least frequent in the extreme group (4.2%). The proportion of female donors was higher in the extreme (45.8%) and moderate (47.2%) groups compared to mild (39.7%) (*p* = 0.017).

Hemodynamic data revealed a decreasing cardiac index with increasing undersizing severity (extreme: 2.49 ± 1.53 vs. mild: 3.09 ± 2.29 L/min/m^2^; *p* = 0.003; Table 1). Other hemodynamic parameters—including pulmonary artery pressure, wedge pressure, pulmonary vascular resistance, and transpulmonary gradient—did not differ significantly (*p* > 0.17). The use of ECMO or VAD as a bridge to transplant was not different. The extreme group enjoyed higher listing status with the older listing status but not with the newer listing status (Table 1). While the donor to recipient hospital distance was not different, the wait-list times was lower in the extreme mismatch group (*p*−0.03).

Survival progressively declined with increasing undersizing: 30-day (87.3% vs. 95.7%), 1-year (79.6% vs. 90.0%), and 5-year survival (69.0% vs. 81.0%) were lowest in the extreme mismatch group (*p* < 0.005; Table 1). Treated rejection at 1 year was more common in this group (21.8% vs. 19.6%; *p* = 0.020), while stroke, pacemaker use, and hospital stay were similar across groups (*p* > 0.10).

### Temporal trends

#### Oversizing

Extreme oversizing increased over time, peaking in 2008–2017 (~ 30%) before a slight decline in 2018–2025. Moderate oversizing followed a similar pattern, while mild mismatches (≤ 20%) remained stable (Supplementary Fig. 1a). Age-wise, extreme oversizing was most frequent in infants (< 1 year; > 20%) and declined with age, whereas mild mismatches were more common in older recipients (11–17 years; ~ 11%) (Supplementary Fig. 2a).

#### Undersizing

Extreme undersizing declined steadily from ~ 40% in 1987–1997 to ~ 14% in 2018–2025. Mild undersizing increased over time (~ 16% to ~ 25%), while moderate undersizing peaked in 2008–2017 (~ 31%) (Supplementary Fig. 1b). Across ages, mild undersizing was most common, especially in recipients aged 11–17 years (~ 35%). Extreme undersizing remained rare, peaking at ~ 2% in infants (Supplementary Fig. 2b).

### Kaplan–Meier survival

**Fig. 2:**
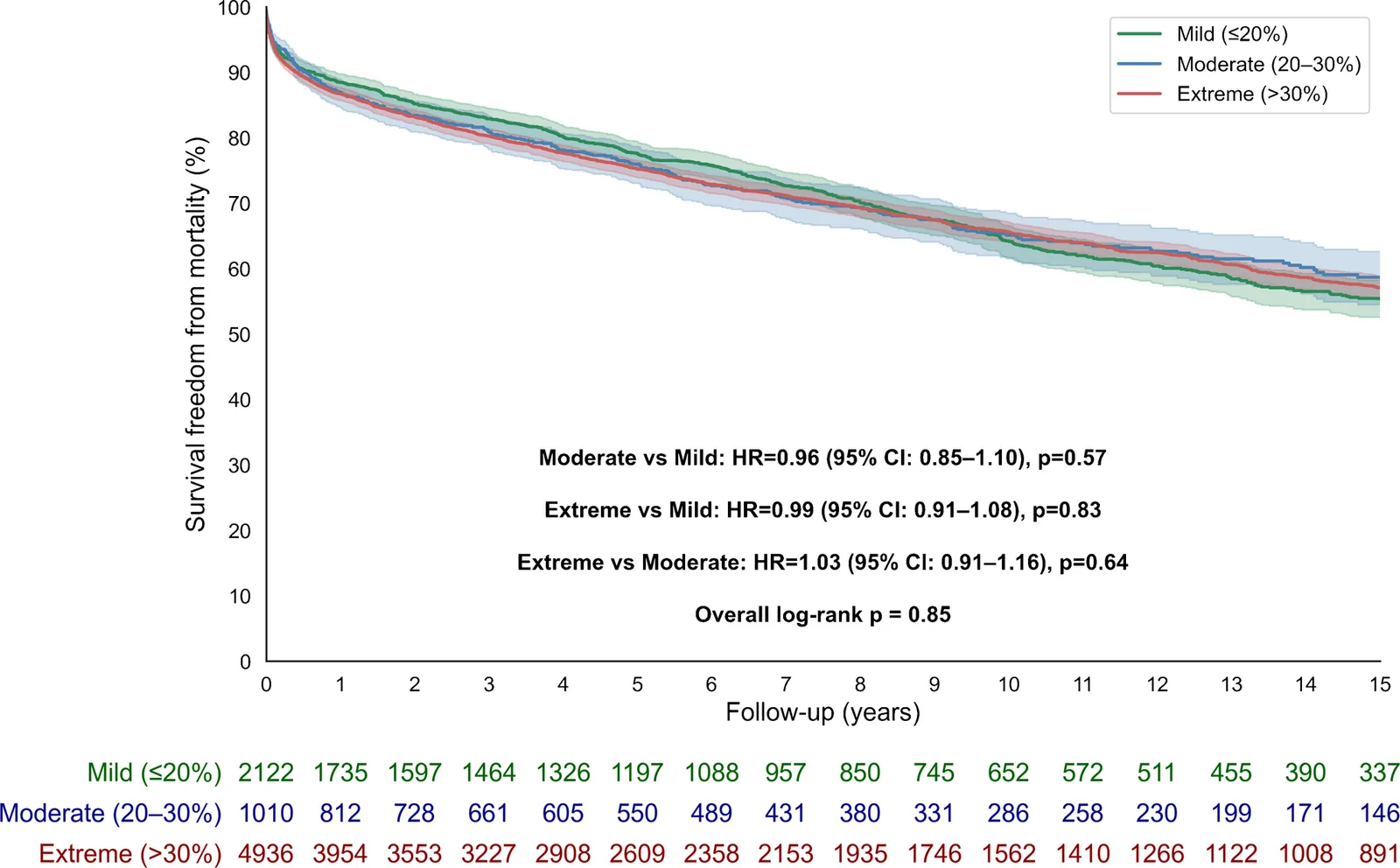
Kaplan Meier curves: 15-year post-transplant survival by oversizing weight disparity group

**Fig. 3:**
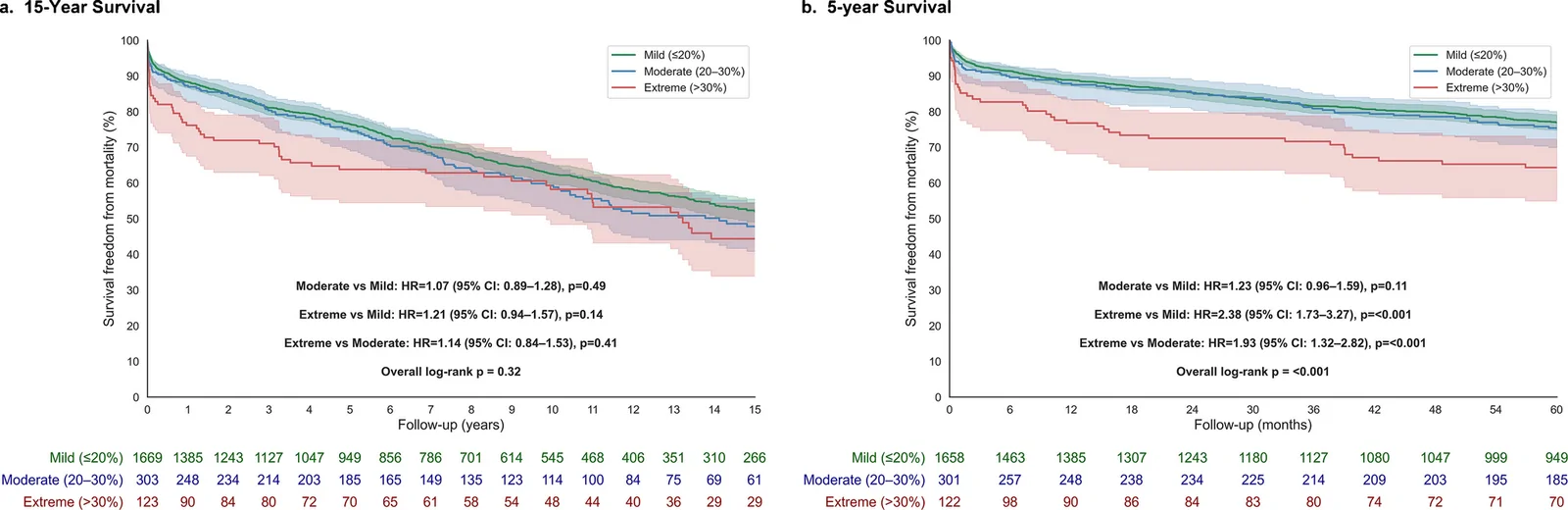
Kaplan Meier curves: post-transplant survival by undersizing weight disparity group

Survival over 15 years did not differ significantly among oversizing groups (log-rank *p* = 0.85), with comparable hazard ratios: moderate vs. mild (HR 0.96, *p* = 0.57), extreme vs. mild (HR 0.99, *p* = 0.83), and extreme vs. moderate (HR 1.03, *p* = 0.64) (Fig. 2). In undersized recipients, long-term survival was also similar across groups (log-rank *p* = 0.32), with no statistically significant differences observed: moderate vs. mild (HR 1.07, 95% CI: 0.89–1.28, *p* = 0.49), extreme vs. mild (HR 1.21, 95% CI: 0.94–1.57, *p* = 0.14), and extreme vs. moderate (HR 1.14, 95% CI: 0.84–1.53, *p* = 0.41) (Fig. 3a). However, 5-year Kaplan–Meier analysis revealed significantly worse early survival in extreme undersizing: survival at 60 months was 81–82% in mild, 64–65% in moderate, and 58–59% in extreme groups (log-rank *p* < 0.001). Early mortality risk was notably higher in extreme vs. mild (HR 2.38, *p* < 0.001) and extreme vs. moderate (HR 1.93, *p* < 0.001), while moderate vs. mild showed a non-significant trend (HR 1.23, *p* = 0.11) (Fig. 3b).

### Mortality predictors

#### Early mortality

**Fig. 4:**
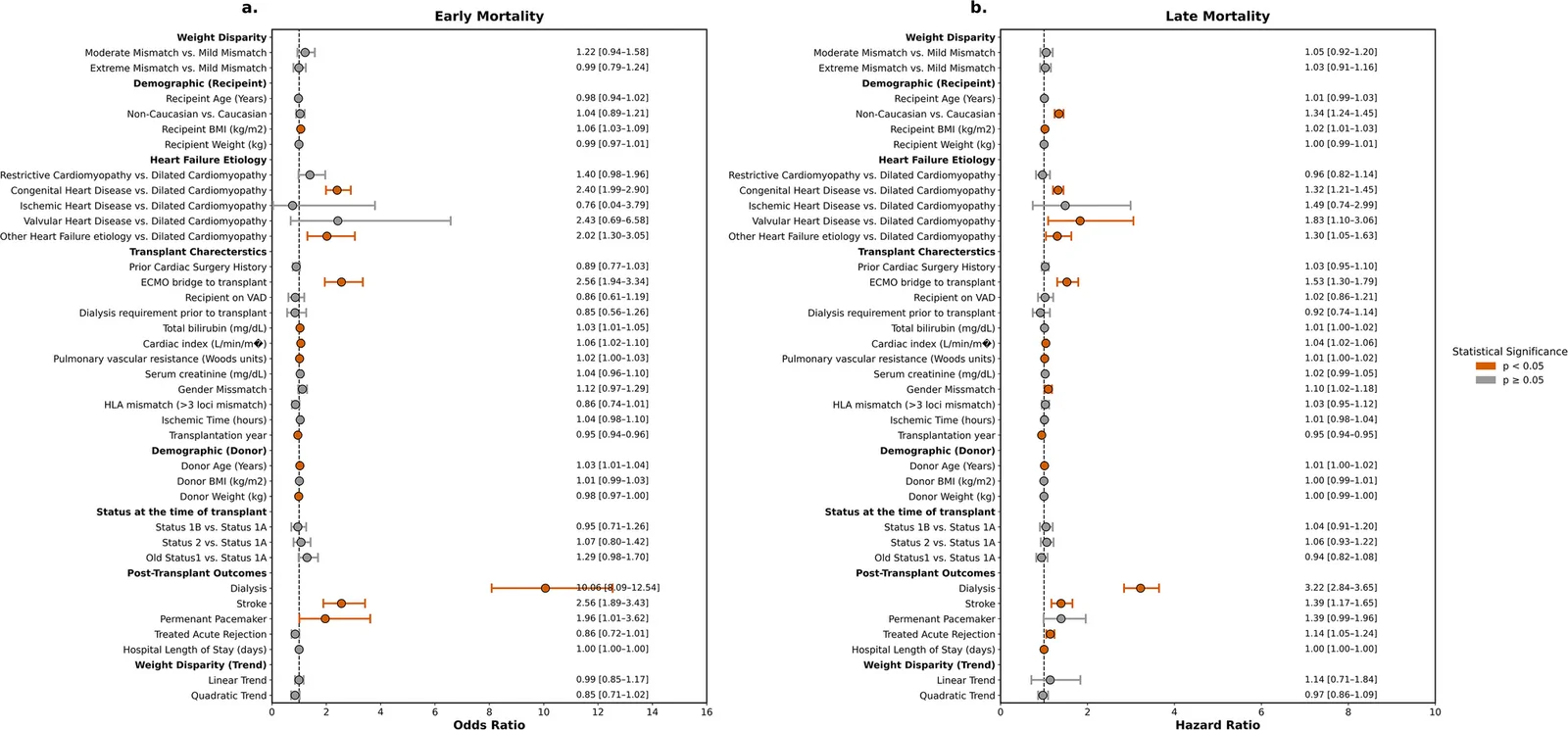
Oversizing group: Predictors of mortality regression analysis forest plot

**Fig. 5:**
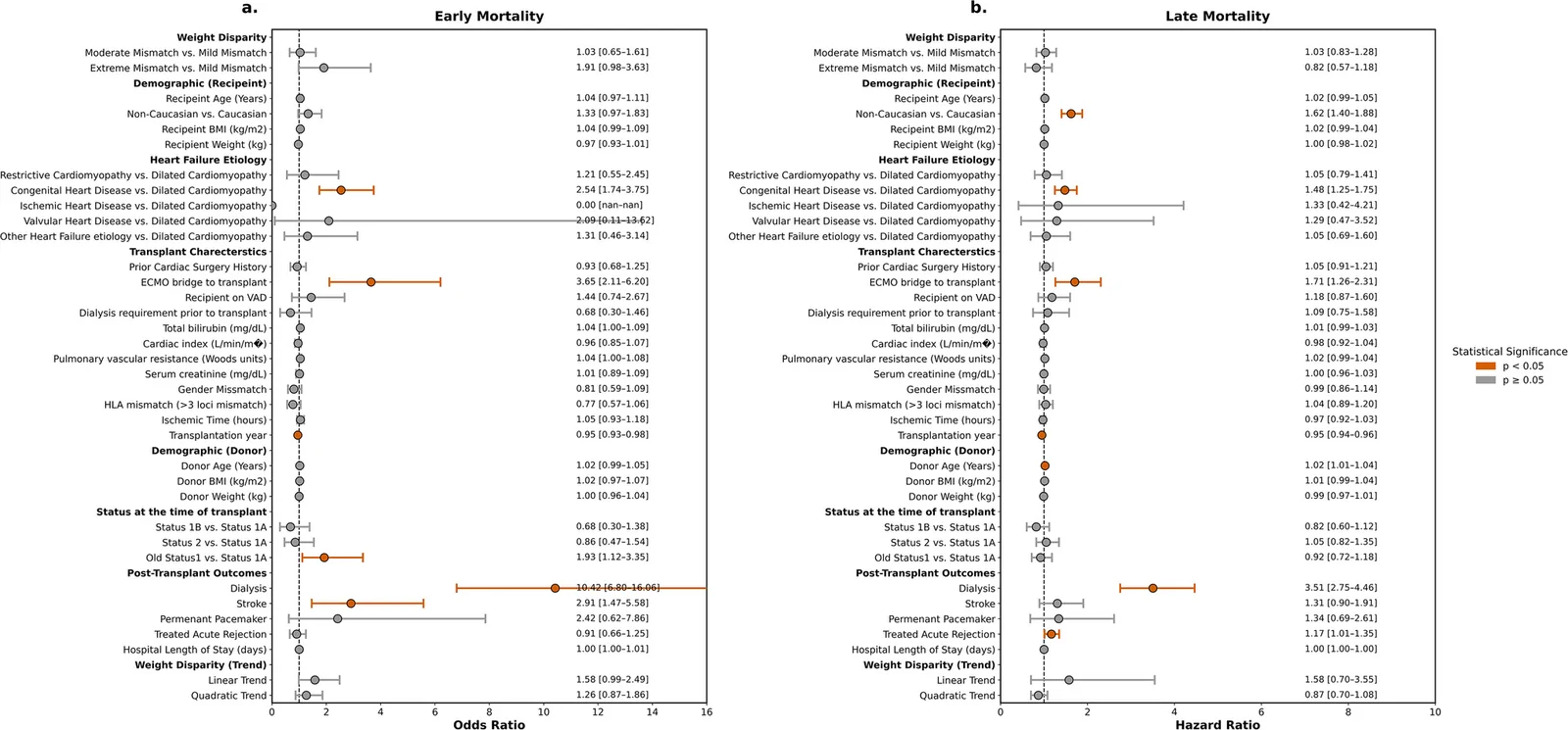
Undersizing group: Predictors of mortality regression analysis forest plot

Multivariable logistic regression models identified key predictors of 1-year mortality in both oversizing and undersizing cohorts (Figs. 4a and 5a). In neither group was weight disparity—whether categorized or modeled as a linear or quadratic trend—significantly associated with early mortality. In the oversizing group, independent predictors included higher recipient BMI, Congenital heart disease, ECMO bridge to transplant [OR: 2.56; 95%CI: (1.94—3.34)], elevated total bilirubin [OR: 1.03; 95%CI: (1.01—1.05)], increased cardiac index [OR: 1.06; 95%CI: (1.02—1.10)] and pulmonary vascular resistance [OR: 1.02; 95%CI: (1.00—1.03)], earlier transplant era [OR: 0.95; 95%CI: (0.94—0.96)], older [OR: 1.03; 95%CI: (1.01—1.04)] and smaller donors [OR: 0.98; 95%CI: (0.97–0.99)], and adverse post-transplant outcomes such as pacemaker requirement [OR: 1.96; 95%CI: (1.01—3.62)], dialysis [OR: 10.06; 95%CI: (8.09—12.54)] and stroke [OR: 2.56; 95%CI: (1.89—3.43)]. Similarly, in the undersizing cohort, significant predictors of early mortality included congenital heart disease [OR: 2.54; 95% CI: (1.74—3.75)], ECMO bridge to transplant [OR: 3.65; 95% CI: (2.11—6.20)], earlier transplant era [OR: 0.95; 95% CI: (0.93—0.98)], older UNOS Status 1 [OR: 1.93; 95% CI: (1.12—3.35)], and adverse post-transplant outcomes such as dialysis [OR: 10.42; 95% CI: (6.80—16.06)], and stroke [OR: 2.91; 95% CI: (1.46—5.58)].

#### Late mortality

Multivariable Cox regression models (Figs. 4b and 5b) revealed no significant association between weight disparity and 15-year mortality in either cohort, regardless of whether it was modeled categorically or continuously. In the oversizing group, independent predictors of late mortality included non-Caucasian race [HR: 1.34; 95% CI: (1.24—1.45)], higher BMI [HR: 1.02; 95% CI: (1.01—1.04)], valvular [HR: 1.83; 95% CI: (1.10—3.06)] and congenital heart failure etiologies [HR: 1.32; 95% CI: (1.21—1.44)], ECMO bridge [HR: 1.53; 95% CI: (1.30—1.79)], elevated bilirubin [HR: 1.01; 95% CI: (1.00—1.02)], increased pulmonary vascular resistance [HR: 1.01; 95% CI: (1.00—1.02)], gender mismatch [HR: 1.10; 95% CI: (1.02—1.18)], earlier transplant era [HR: 0.95; 95% CI: (0.94—0.95)], older donors [HR: 1.01; 95% CI: (1.00—1.02)], post-transplant dialysis [HR: 3.22; 95% CI: (2.84—3.65)], treated acute rejection history [HR: 1.14; 95% CI: (1.05—1.24)], and stroke [HR: 1.39; 95% CI: (1.17—1.65)]. In the undersizing group, significant predictors included non-caucasian race [HR: 1.62; 95% CI: (1.40—1.88)], congenital heart disease [HR: 1.48; 95% CI: (1.25—1.75)], ECMO bridge [HR: 1.71; 95% CI: (1.26—2.31)], earlier transplant era [HR: 0.95; 95% CI: (0.94—0.96)], older donors [HR: 1.02; 95% CI: (1.01—1.04)], and adverse post-transplant outcomes such as dialysis [HR: 3.51; 95% CI: (2.75—4.46)], and acute rejection treatment history [HR: 1.17; 95% CI: (1.01—1.35)]. Weight disparity—whether modeled as a linear or quadratic trend—did not demonstrate a statistically significant effect on long-term mortality.

## Discussion

Body weight is commonly used as a surrogate to represent cardiac mass and size, particularly in the donor where normal anatomical and physiological constraints are expected to be present [4, 5]. Hence, weight based matching of donor to recipient is still the most commonly used method in pediatric heart transplantation. This is despite other methods such as volumetric matching based on cross sectional imaging such as using computed tomography or various nomograms based on a combination of height, age, gender and weight metrics and is due to familiarity and consistent availability of weight as a metric both in the donor and recipient and the converse in the other methods of matching as previously alluded [6, 7].

Of the 11,583 patients transplanted during the study period, 9175 (80%) had donor oversizing with 62% having extreme donor weight oversizing (> 30% mismatch) (Fig. 1). Donor to recipient oversizing is used when the recipient heart size is larger than expected based on recipient body weight and size such as in dilated cardiomyopathy where a larger heart from a bigger donor can be accommodated, thus expanding the donor pool and helping with the wait-list times [10, 11]. This was seen in this study where wait-list times were lower with increasing size mismatch (Table 1).

Donor to recipient oversizing is also used when an increased physiological demand is expected in the recipient. This could be due to higher afterload from increased pulmonary vascular resistance or the need for increased cardiac out output due to wastage from aortopulmonary collaterals as seen as in CHD, especially of the cyanotic type [12]. This was reflected in this study by the higher prevalence of primary cardiac diagnosis of CHD in the extreme oversizing group as was the presence of elevated cardiac output and higher pulmonary vascular resistance noted in the hemodynamic data in this group, reflecting the need for oversizing (Table 1).

The extreme oversizing group recipients were also younger in age. This is likely from correlation of CHD as the most common cause for cardiac transplantation in this age group and the need to oversize based on diagnosis as previously alluded [12]. Besides, the probability to oversize is higher with recipients of lower weight and age due to a larger donor pool with increasing age and hence weight in pediatric transplantation. Further, the increase in donor to recipient hospital distance and its resulting increase in the ischemic times seen in extreme oversizing group further supports the idea of oversizing as a measure to expand the donor pool in the younger age recipients and the resultant decrease the wait-list times with increasing extent of oversizing as seen in this study (Table 1).

Another reason for oversizing is the presence of a VAD as a bridge to transplantation. The VAD, besides expanding the mediastinal space, may justify oversizing due to the occurrence of elevated pulmonary vascular resistance [13]. However, the use of VAD was actually lower in extreme oversizing group and likely reflects the younger age group where VAD utilization and successful outcomes of a bridge to transplantation are generally lower [14].

Post transplant survival was similar both in the short-term and long-term till 15-years of follow-up in the oversizing group. Lack of effect of oversizing on post-transplant survival was further confirmed using multivariable models for at 1- year and 15-years survival. Previously described negative prognostic factors such as CHD diagnosis, ECMO bridge, elevated PVR, and occurrence post operative complications such need for dialysis and stroke were predictive of short-term and long-term mortality and not oversizing [4, 15, 16]. Extreme oversizing, defined as > 30% donor to recipient weight mismatch occurred in 62% of the patients in this study. This again suggests that oversizing, by cutting down wait-list times due to donor pool expansion, especially in the younger patients where donor scarcity is more likely may be only limited by the ability of the recipient mediastinal volume to accommodate the donor heart without any adverse consequence due to the mismatch on post-transplantation survival [12].

Interestingly, the treated rejection in the first year was lower in the extreme oversizing group. This likely due to the younger age of the recipient with higher immune tolerance. This was further corroborated by higher use of ABO transplant in this group. In addition, the extent of Human Leukocyte Antigen (HLA) mismatch was lower in this group [16].

As compared to oversizing, undersizing was less commonly utilized at 20% (2408) of the 11,583 patients transplanted in the study. Of this, only 6% were of the extreme undersizing group (> 30%).

The extreme undersizing group were less likely to have restrictive cardiomyopathy as the underlying diagnosis. While a smaller recipient heart size for a given body weight in restrictive cardiomyopathy maybe conducive for undersizing, the increased association of elevated pulmonary vascular in restrictive cardiomyopathy generally prevents such undersizing and may have been a plausible cause in this study [16]. While the transpulmonary gradient was higher in the extreme mismatch group in this study, it did not reach statistical significance (Table 1).

**Fig. 6:**
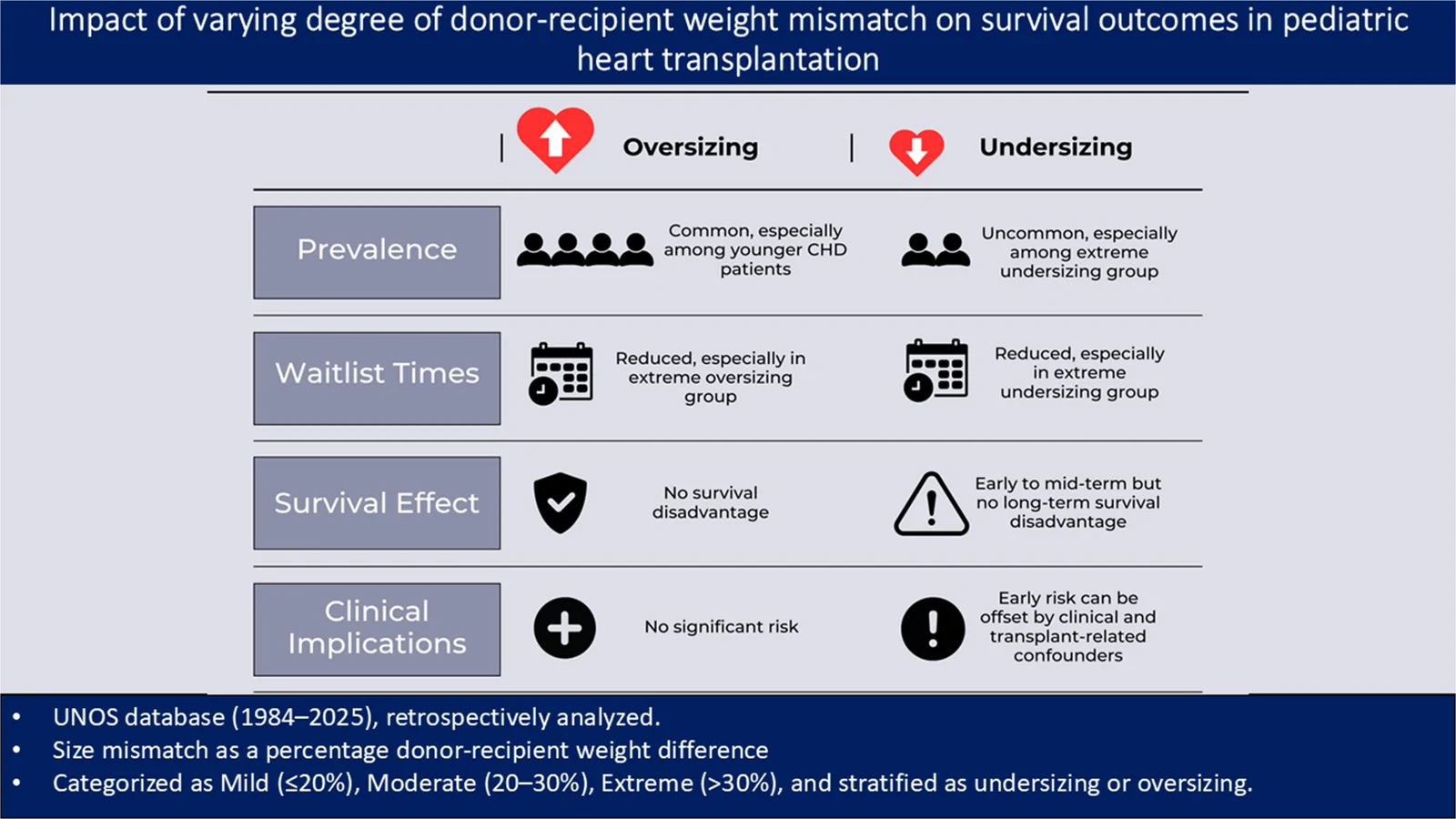
Implications of donor-recipient size matching in pediatric heart transplantation

The undersizing group also had a lower cardiac listing status with the newer listing status as UNOS Status 2. Thus undersizing could have been a measure to increase the donor pool especially in this group. This was reflected by the decreased waitlist times in the extreme in oversizing group (Table 1). The 30-day, 1 year and 5-year survival was low, particularly in the extreme undersizing group (Table 1, Fig. 3). However, a follow-up to 15 years did not show a survival difference. The early survival penalty may reflect physiologic mismatch in select recipients, especially those with elevated pulmonary vascular resistance or higher transpulmonary gradients, where a smaller allograft could have limited hemodynamic reserve. Over time, graft adaptation may mitigate this disadvantage, resulting in convergence of survival curves in the long term [17, 18]. However, as seen in multivariable analysis, the only independent predictors of short and long-term mortality were CHD diagnosis, elevated pulmonary vascular resistance, occurrence of elevated pulmonary vascular resistance, older era of transplantation and occurrence of post-transplant complication such as dialysis use, stroke and treated rejection suggesting these were stronger drivers of a negative survival outcomes than undersizing itself (Fig. 6) [4, 15, 16].

### Limitations

The retrospective nature of this study and its dependence on the UNOS registry, which provides robust follow-up data at the national level but lacks specific clinical variables like imaging data, perioperative management, and surgical technique, are its main limitations. Besides clinical data such as mediastinal compromise such as airway compression, inability to close the chest, and cardiac compression after implantation of an oversized allograft are not available. Similarly, clinical considerations that may support the use of an undersized donor allograft such as precise pressure volume relationships, right-sided loading conditions, or individualized hemodynamic tolerance are not recorded. The absence of postoperative imaging further limits assessment of graft adaptation over time, precluding evaluation of ventricular remodeling in either oversizing or undersizing scenarios. Although DRWR is a universally accessible and clinically pragmatic metric, it fails to capture the anatomical or physiological subtleties of size matching. More precise volumetric assessments, such as estimated total cardiac volume (eTCV), provide enhanced physiological significance; however, they are impractical for extensive datasets due to the lack of standardized imaging data. Finally, unmeasured confounding, including center-level practices such as bias on donor preference based on weight might exist may limit generalizability and bias outcome associations.

## Conclusion

Oversizing of the donor to the recipient is more common in pediatric heart transplantation than undersizing. Oversizing, especially extreme oversizing is more common in younger age recipients with congenital heart disease. Overall oversizing has no impact of short-term and long-term survival post transplantation. Undersizing was uncommonly used in pediatric transplantation, particularly extreme undersizing. While undersizing showed early to mid-term but not long-term survival disadvantage, this disappeared when corrected for factors such as underlying cardiac diagnosis, presence of elevated pulmonary vascular resistance, transplantation era, and occurrence of post-transplant complications such as stroke, rejection and dialysis use.

## Supplementary Information

Supplemental Figure 1 Era trends of weight disparity among heart TX. (PNG 111 kb)

High Resolution Image (TIFF 952 KB)

Supplemental Figure 2 Trends of weight disparity among Heart TX per Age group. (PNG 82.3 kb)

High Resolution Image (TIFF 677 KB)

Supplemental Figure 3 (PNG 91.3 KB)

Supplementary file3 (MP4 11791 KB)

## Data Availability

This study utilized data from the United Network for Organ Sharing (UNOS) registry. The dataset is publicly available upon application to UNOS/OPTN. All relevant derived data are presented within the article and Supplementary Materials.
