# Supplementary figures and images for: Impact of varying degree of donor-recipient weight mismatch on survival outcomes in pediatric heart transplantation
Source: Indian J Thorac Cardiovasc Surg. 2026 Feb 28;42(7):831–43. doi: 10.1007/s12055-026-02195-8 (PMC13305213; doi:10.1007/s12055-026-02195-8)

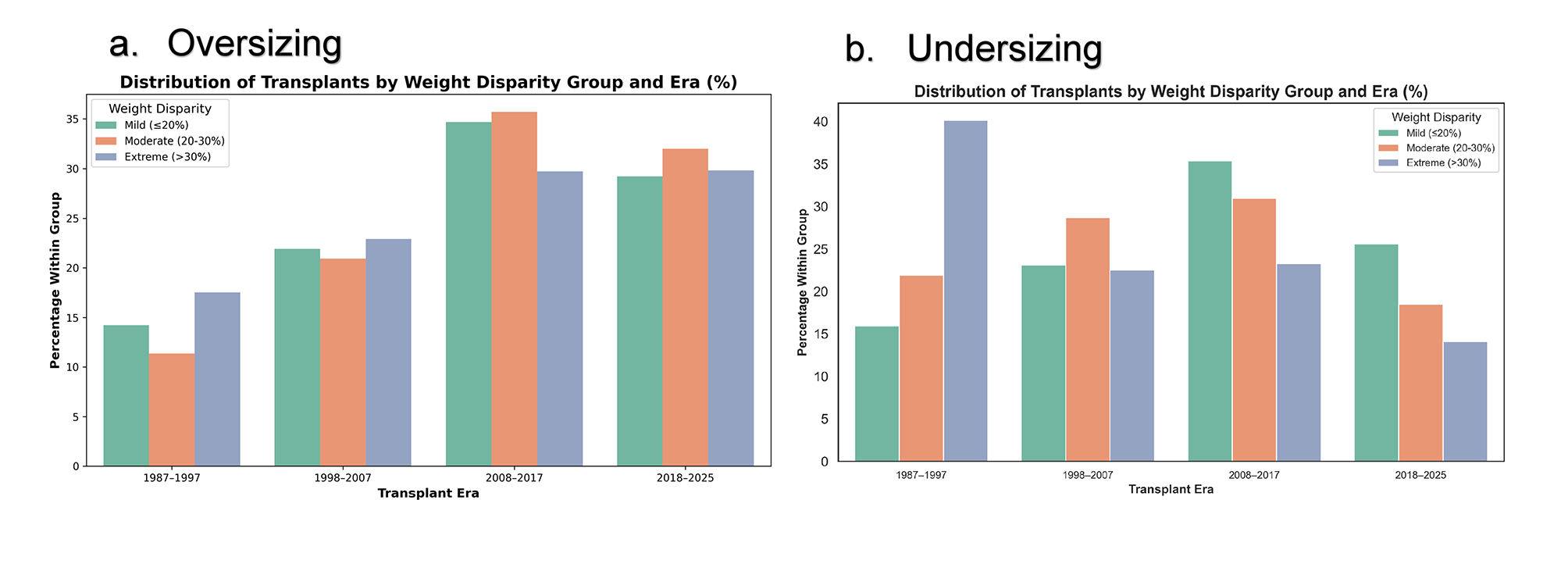

Supplement: Supplementary file 1 — Era trends of weight disparity among heart TX. (PNG 111 kb) [file 12055_2026_2195_Fig7_ESM.png]

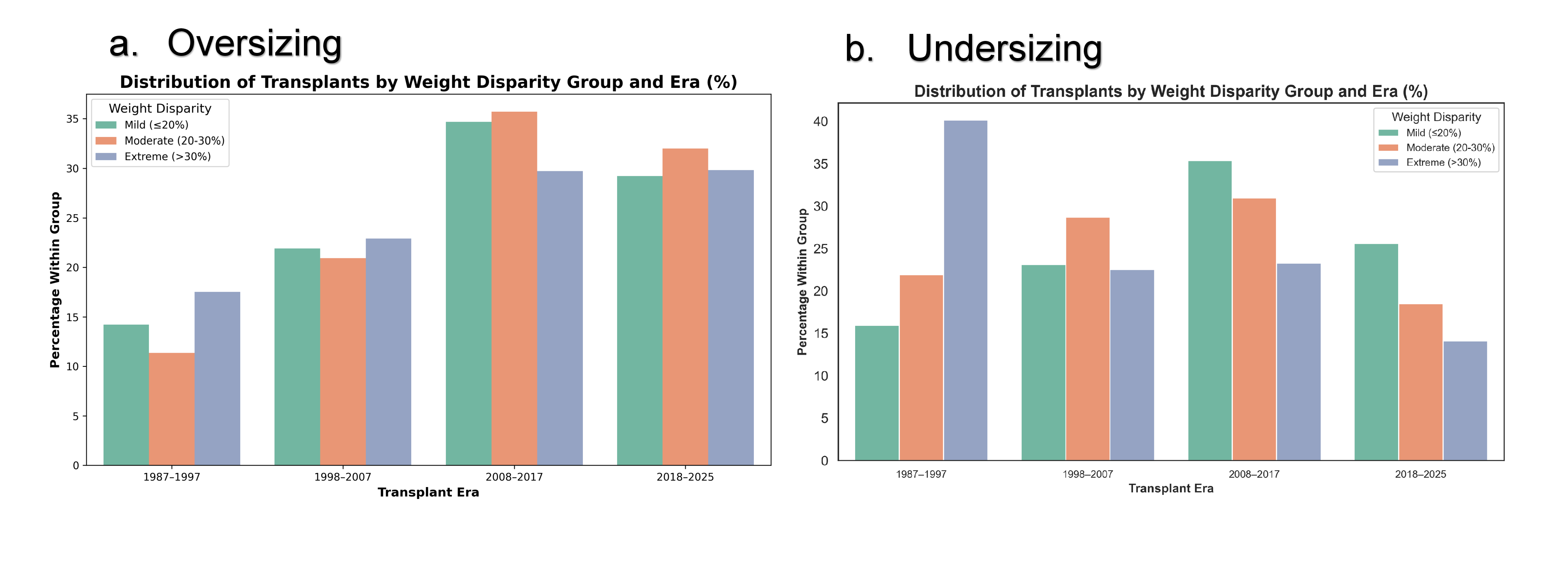

Supplement: Supplementary file 2 — High Resolution Image (TIFF 952 KB) [file 12055_2026_2195_MOESM1_ESM.tiff]

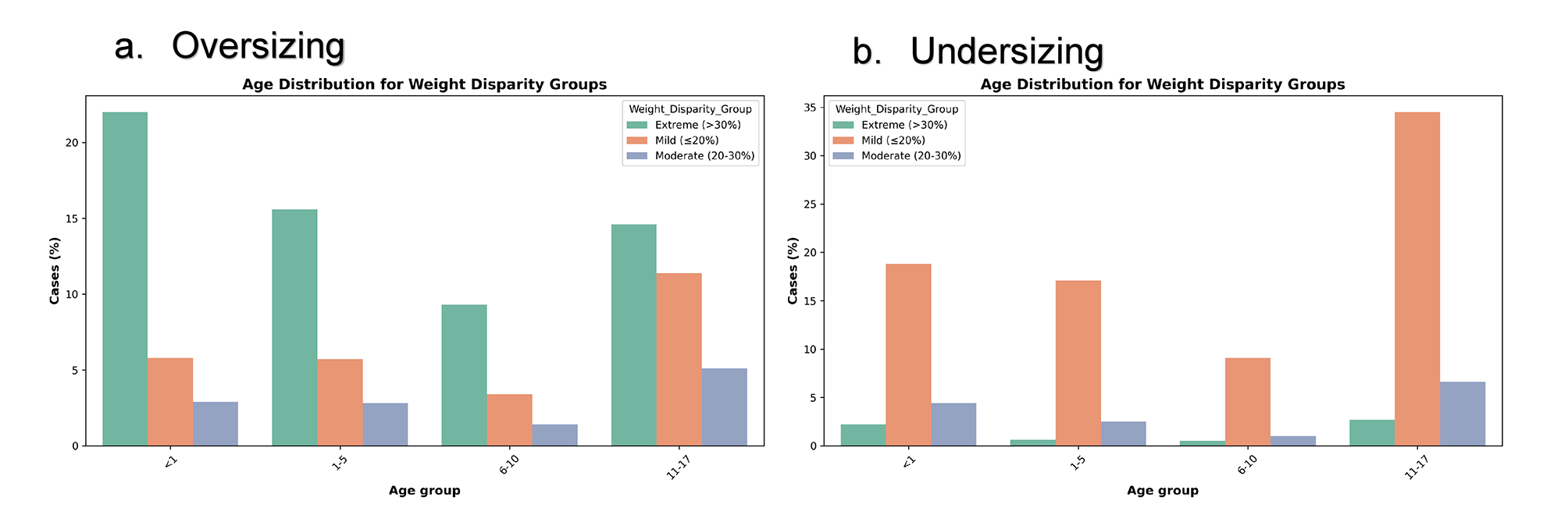

Supplement: Supplementary file 3 — Trends of weight disparity among Heart TX per Age group. (PNG 82.3 kb) [file 12055_2026_2195_Fig8_ESM.png]

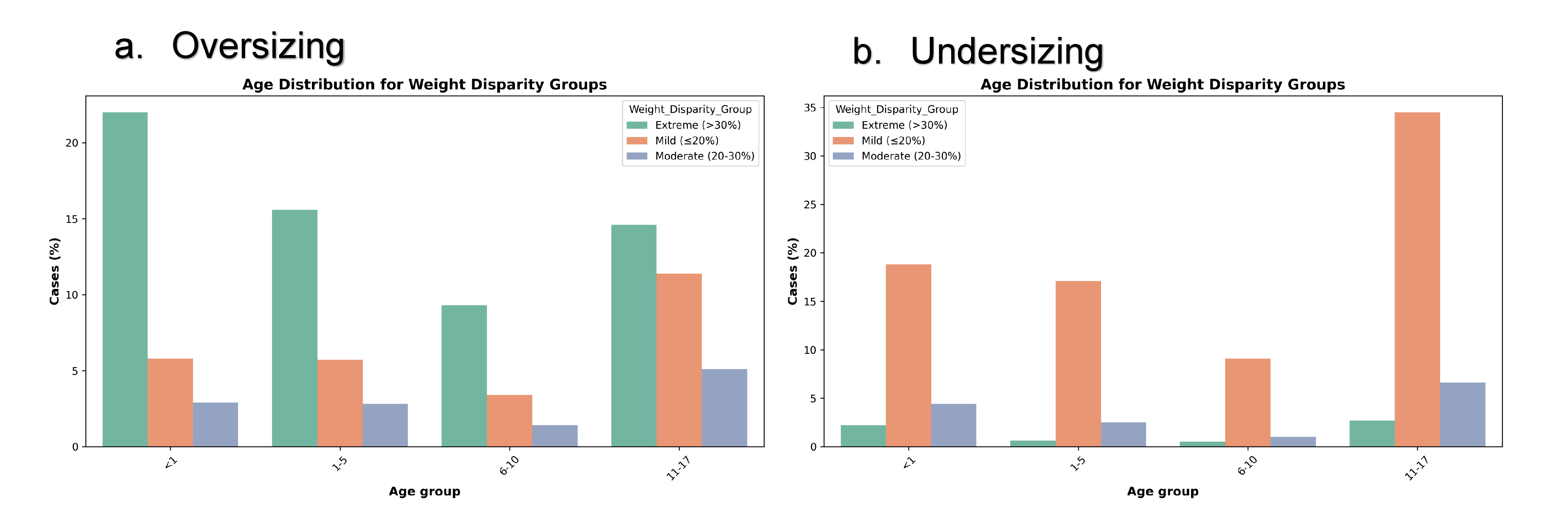

Supplement: Supplementary file 4 — High Resolution Image (TIFF 677 KB) [file 12055_2026_2195_MOESM2_ESM.tiff]

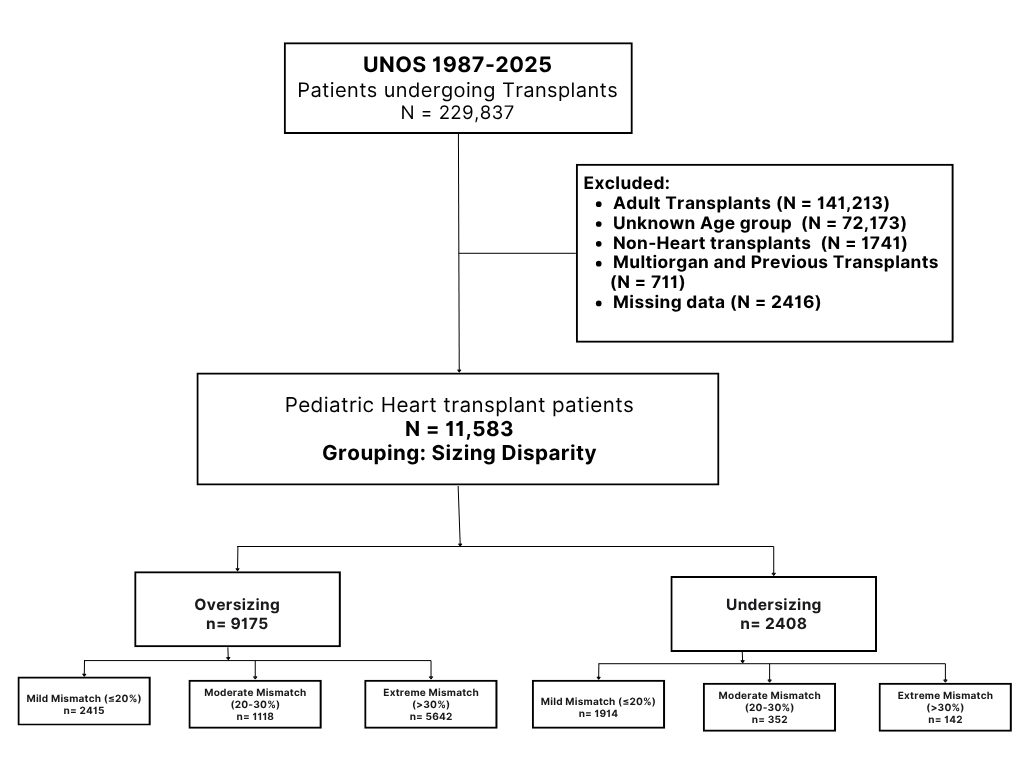

Supplement: Supplementary file 5 — (PNG 91.3 KB) [file 12055_2026_2195_MOESM3_ESM.png]
